# Czech Adolescents’ Face-to-Face Meetings With People from the Internet: The Role of Adolescents’ Motives and Expectations

**DOI:** 10.1007/s10964-022-01697-z

**Published:** 2022-11-08

**Authors:** Vojtěch Mýlek, Lenka Dedkova, Gustavo S. Mesch

**Affiliations:** 1 Interdisciplinary Research Team on Internet and Society, Faculty of Social Studies, Masaryk University, Joštova 10, Brno, 60200 Czech Republic; 2 Department of Sociology, Faculty of Social Sciences, University of Haifa, 199 Aba Khoushy Ave. Mount Carmel, Haifa, 3498838 Israel

**Keywords:** Adolescence, Online relationships, Face-to-face meetings, Motives, Expectation disconfirmation, Social compensation

## Abstract

Research of face-to-face meetings between adolescents and people met online stands on untested assumptions that these meetings are uniform, and adolescents attend them to expand their social circle. It is also unclear what makes such meetings pleasant or unpleasant. This study examined meetings of 611 Czech adolescents (age 11–16, *M*_*age*_ = 14.04, *SD* = 1.67, 47.1% female). Face-to-face meetings attended with friendly, romantic, or instrumental motives differed from each other, emphasizing the need to investigate them separately. Pleasantness of meetings is closely related to disconfirmation of adolescents’ expectations. Unmet expectations related to unpleasant meetings, exceeded expectations to pleasant ones. While present findings uphold existing theories (e.g., social compensation), they also call for new theoretical perspectives for this common adolescents’ activity.

## Introduction

Adolescents commonly make new social connections online. Sometimes they meet these new contacts face-to-face. Such in-person meetings with people met online may present valuable opportunities to fulfill adolescents’ developmental needs (e.g., to form a new social or romantic relationship; Pascoe, 2011), but it may also expose youth to significant risks (e.g., sexual assault; Priebe & Svedin, 2012). Given the popularity of these meetings and the high concerns regarding their impact on adolescents’ well-being, many studies have focused on the correlates of this activity. These studies share a crucial limitation – they treat all face-to-face meetings as a uniform phenomenon and overlook the differences in adolescents’ motivations for attending them, or the length of the online interaction. Such an approach yields a limited picture of the diverse set of situations that may vary in a number of aspects, including how risky or beneficial the meetings may be (e.g., meeting with a longtime online friend vs. a spontaneous date after a quick chat on Tinder). It also raises the question of the appropriateness of the theoretical explanations. Moreover, the interest in this topic is largely driven by public and media panic under the assumption that these meetings are generally risky, but only a few studies have considered adolescents’ own reflections of how pleasant or upsetting the experience actually was. While most European adolescents reported feeling happy about their most recent face-to-face meeting with online friends (Smahel et al., 2020), it is not clear which meetings are more likely to be pleasant and which are more likely to be upsetting. The current study addresses these gaps in two ways. First, it categorizes face-to-face meetings based on adolescents’ motives to attend them and investigates how the various reasons differ in several key aspects. Second, the study investigates the factors that relate to whether the offline face-to-face meeting was pleasant, upsetting, or a neutral experience for the adolescent. The study aims to contextualize the existing findings about adolescents’ face-to-face meetings, to provide input for their re-conceptualization in future research, and to suggest theoretical frameworks that are adequate for their examination.

### Who Attends Face-to-Face Meetings and Why?

In a recent survey, between 23% (Italy) and 57% (Norway) of European 9–16-year-olds reported that they have been in online contact with someone they had not met in person. Meeting these online acquaintances in person is less common, but not unusual — between 5% (France) and 25% (Serbia) of adolescents reported going to an offline, face-to-face meeting with someone from the internet in the past year (Smahel et al., 2020). The general public’s perception of children and adolescents’ meetings with people from the internet are closely connected to fears of sexual assault. The possibility that a child becomes the victim of “cybergrooming” consistently ranks at the top of parents’ internet-related concerns (e.g., Auxiere et al., 2020). The potential risks of face-to-face meetings are commonly exaggerated in the media and public debates (see Mýlek et al., 2021). This is reflected in the existing research, which often adopts a risk-focused perspective. Previous studies commonly conceptualize face-to-face meetings as risk-taking behavior and focus on the examination of which adolescents are more likely to attend them (e.g., Bayraktar et al., 2016; Marret & Choo, 2018) or on the assessment of the prevalence of risky meetings and their adverse outcomes (e.g., Greene-Colozzi et al., 2020; Priebe & Svedin, 2012).

The first of the two abovementioned research streams has identified many factors associated with the likelihood that adolescents will go to a face-to-face meeting. The adolescents who attend these meetings tend to be older, have higher self-efficacy, sensation seeking, or more psychological difficulties, and use the internet more (e.g., Mýlek et al., 2020). They view more online pornography and disclose more personal information online (Marret & Choo, 2018). They have higher digital skills, feel more confident online, and value the anonymity of their online communication (Livingstone and Helsper, 2007). Going to such a meeting once increases the chances of going again (Van den Heuvel et al., 2012). On the other hand, the lower likelihood of attending a face-to-face meeting is associated with more parental supervision or restrictions (e.g., Hasebrink et al., 2011), a higher quality of parent-child communication, higher shyness, and higher loneliness, but also higher self-esteem (Van den Heuvel et al., 2012) and higher life satisfaction (Livingstone & Helsper, 2007). These studies illuminate the adolescents who attend these meetings but do not offer much information on what kinds of meetings they attend or why. Consequently, it is tricky to interpret their results and connect them to specific behaviors (e.g., meeting friends, dating). Some findings also seem contradictory (e.g., the positive effects of both shyness and self-efficacy), which may indicate that they relate to different kinds of face-to-face meetings.

Despite rarely being explicitly described, the kind of meeting is often implicitly assumed in the previous research. While many of the studies do not draw on one specific theory, several use the *social compensation hypothesis* to justify their arguments (e.g., Mýlek et al., 2020) or interpret their results (e.g., Bayraktar et al., 2016). This hypothesis proposes that adolescents who struggle to form supportive and fulfilling social relationships offline (because of their social anxiety or other inhibiting factors), will turn to the internet to compensate for their unsatisfied needs for social connections (see Mesch, 2019). In contrast, *the rich-get-richer hypothesis* (also labeled as “social enhancement hypothesis”) proposes that adolescents who are more socially apt offline (e.g., more extraverted) will use the internet to further extend their social network and they are more likely to form new social relationships online (see Mesch, 2019). Thus, studies that invoke these hypotheses essentially assume that face-to-face meetings are usually attended for social motives, like to form new relationships. The assumption is not unfounded because both the social compensation and rich-get-richer hypotheses garnered empirical support (e.g., Valkenburg and Peter, 2007). However, it remains necessary to directly test whether adolescents meet people from the internet predominantly to form new social connections or whether there are other motives at play (and how prevalent they are).

Research to investigate the motivations for “cyber relationships” shows that interactions with unknown people (i.e., chatting online with unknown people, using dating apps, making online friends) can be driven by a variety of motives, including escapism, entertainment, romance seeking, and social approval (Peter et al., 2006; Timmermans & De Caluwé, 2017; Wang & Chang, 2010). However, none of these studies directly focused on in-person meetings and only one focused on adolescents (Peter et al., 2006). To the authors’ knowledge, only one qualitative study asked adolescents about their motives for attending face-to-face meetings with people they met online (notably, the study exclusively examined upsetting meetings). Adolescents reported two main motives – going on a romantic date and making a new friend (Dedkova et al., 2014). Both activities can lead to the establishment of a new relationship and fall under “social motive”, but they differ in the type of desired relationship. This can affect aspects of the meeting (e.g., how long adolescents talk to the person online before meeting them offline) and the selection of the person to meet. Adolescents may also meet with others for pragmatic reasons (e.g., to buy/sell/swap something). Such instrumental meetings likely differ from those focused on forming a relationship. For instance, adolescents may put less effort into getting to know the person before the meeting. From a theoretical standpoint, these kinds of instrumental meetings would also fall outside the above-mentioned hypotheses that drive the current research in online interactions (i.e., social compensation, rich get richer) and they would unlikely be explained by the individual characteristics typically examined under these theoretical lenses (e.g., shyness). Therefore, the motives for attending the meeting (i.e., friendly, romantic, instrumental) may provide a useful way to categorize different kinds of face-to-face meetings. This also allows for a more specific evaluation of which meetings are more likely to be pleasant or unpleasant for adolescents. Notably, an unpleasant meeting does not have to be risky or harmful. Likewise, what is reported as a pleasant meeting by the adolescent does not have to be safe and it can include engagement in a number of risk-taking behaviors. However, considering adolescents’ reflections of which meetings were (un)pleasant for them can be the first step towards better-directed prevention and safety advice.

### Pleasant and Unpleasant Face-to-Face Meetings

Although researchers agree that the interactions with people from the internet can be both beneficial and potentially risky for adolescents (e.g., Smahel et al., 2020), the existing research is dominantly focused on the negative impacts. Since the fears about potential harm usually revolve around the possibility that the adolescent will be assaulted (sexually or otherwise) by the person they meet (Auxiere et al., 2020), previous studies often focused on the prevalence of such an assault. Two Scandinavian studies of older adolescents suggest that the rates of sexual assault are low, though not negligible. In both, approximately 6% of the adolescents (ages 14–17 and 16–22) who went to a face-to-face meeting reported some sexual assault (Helweg-Larsen et al., 2012; Priebe & Svedin, 2012). A wider pan-European study shows that 11% of youth from across Europe (i.e., approximately 1% of all of the sampled children and adolescents aged 9–16) reported feeling *bothered* after a face-to-face meeting. Among them, 22% reported verbal harm, 11% sexual harm, and 3% physical harm. Notably, most youth who were bothered did not report any of these – 64% reported something else happened or provided no answer (Livingstone et al., 2011). Results from the United States also suggest that unpleasant meetings are uncommon (Dowdell, 2011). These studies come with important limitations. First, they are more than a decade old, and their results might be outdated. Second, they do not ask about positive experiences. Among more recent studies, EU Kids Online IV more broadly examined adolescents’ own reflections of the meetings. The results indicate that most European children and adolescents (aged 9–16) who went to a face-to-face meeting reported feeling happy about it (between 52% in Slovakia and 86% in Romania), and less than 5% in all of the countries, except for France (11%), reported feeling fairly or very upset (Smahel et al., 2020). While the findings are reassuring, the study does not examine the kinds of face-to-face meetings that are more likely to be upsetting or have positive experiences for adolescents.

### Mismatched and Exceeded Expectations

To the authors’ knowledge, no study has quantitatively examined which factors determine how pleasant the meeting will be for an adolescent. Qualitative results suggest that adolescents have negative feelings about the meeting when the other person was not who they expected (Dedkova et al., 2014). This is in line with the *expectation-disconfirmation theory*, which was originally developed to explain consumer satisfaction (Oliver, 1980). The theory proposes that consumers compare their pre-purchase expectations to the outcomes of their purchase of a product or a service. *Disconfirmation* happens when the reality does not match the expectations – it can be either negative (i.e., an outcome, like the purchased product, is worse than expected), or positive (i.e., the expectations are exceeded). Positive disconfirmation is thought to cause consumer satisfaction, while negative disconfirmation leads to dissatisfaction (Pizam & Milman, 1993). More recently, the theory has been used in various fields to investigate things like trust in technology (Lankton et al., 2016) and satisfaction with remote work (Carraher-Wolverton, 2022).

While the expectation-disconfirmation theory is not usually used in the context of interpersonal interactions, its central propositions are relevant for investigating which face-to-face meetings are pleasant or unpleasant for adolescents. The online communication, which precedes the meeting, allows for careful control of one’s anonymity and self-presentation (e.g., Nesi et al., 2018). This is at the core of the fears about “online predators” who can mislead children and adolescents by pretending to be their peers. The rate at which such deception occurs varies across studies. Among Malaysian adolescents (aged 12–18) who met with someone from the internet, 26.7% met with an adult when they expected a peer (Marret & Choo, 2018). Out of Dutch adolescents (aged 11–18) who went to a face-to-face meeting, only 3.8% met with someone who lied about their identity (Van den Heuvel et al., 2012). Demographics are also not the only source for mismatched expectations. A qualitative study shows that female adolescents’ negative feelings about face-to-face meetings stemmed from deviations from expected appearance (i.e., lower physical attractivity) and behavior (e.g., unwelcomed attempts at physical contact, overconfidence, aggressiveness) (Dedkova et al., 2014). It is important to note that such deviations do not always have to be caused by malicious intentions, nor do they always need to be negative (i.e., the person who comes to the face-to-face meeting can also exceed the adolescent’s expectations). Based on the expectation-disconfirmation theory, positive disconfirmation should make the meeting more pleasant, and negative disconfirmation more unpleasant.

The same qualitative study also pointed out an additional source for negative feelings: the participants reflection of their own behavior. They reported feeling remorse, guilt, or anger toward themselves, and they were unsatisfied with how they handled the meeting, especially if they were not able to stand up for themselves when the other person behaved inappropriately (Dedkova et al., 2014). Thus, social self-efficacy, which reflects adolescents’ confidence in their ability to handle social challenges (Muris, 2001), can be another important predictor for the meeting evaluation. Adolescents with higher social self-efficacy find it easier to initiate small talk, make new friends, and express disagreement (Muris, 2001). Thus, even when the other person has no malicious intentions and behaves in line with expectations, self-efficacy can co-determine the course of the meeting and make it more pleasant.

## Current Study

The existing research of in-person, face-to-face meetings between adolescents and people they met on the internet does not differentiate between the different possible types of these meetings. This reduces the phenomenon, complicates the interpretation of the results, and raises questions about the adequacy of the commonly applied theories. The current study aims to address this gap by categorizing face-to-face meetings based on whether adolescents attended them with friendly, romantic, or instrumental motives. To test if this categorization is meaningful and to investigate how various kinds of meetings differ from each other, the categories of the meetings are compared in several key factors – which adolescents attended them (i.e., gender, age); how long they were in contact online before meeting offline; who they met (i.e., gender of the person, congruence of their age, behavior, appearance with adolescents’ expectations); how afraid they were of getting harmed; and whether they stayed in touch after the meeting. The second aim of this study is to investigate the factors that relate to the (un)pleasantness of the face-to-face meeting. Based on the expectation-disconfirmation theory, negative disconfirmation of adolescent’s expectations (i.e., meeting someone of different-than-expected age or gender, or someone who looks or behaves worse than expected) should make the meeting more likely to be unpleasant. On the other hand, positive disconfirmation (i.e., meeting someone who looks or behaves better than expected) should make the meeting more likely to be pleasant for adolescents. Since expectation disconfirmation may depend on the length of online contact (i.e., longer contact may be conducive to more accurate expectations), this factor is controlled for in the analysis. It is also expected that higher social self-efficacy makes pleasant meetings more likely because it can help adolescents handle difficult or awkward situations.

## Methods

### Participants

The current study uses data from a survey of 2,500 Czech adolescents (age 11–16, *M*_*age*_ = 13.43, *SD* = 1.70; 50.0% female) that were collected in June 2021 as the first wave of a wider three-wave longitudinal study to investigate various aspects of the use of digital technologies and the well-being of adolescents and their parents.

The current study examines adolescents who experienced a face-to-face meeting with someone from the internet in the preceding two years. This timeframe was set for two reasons. First, this includes meetings from before the COVID-19 pandemic, when contact restrictions and potential fears of infection might have led to less meetings and, in turn, a small sample size. Second, it excludes meetings that happened too long ago, because a prolonged amount of time since the experience may cause inaccurate recall. From the original sample, 1,643 (65.7%) adolescents reported no face-to-face meetings and 93 (3.7%) did not provide an answer. Among those who reported attending at least one meeting (*n* = 764), 66 (8.6%) experienced their last meeting more than two years ago (87, 11.4% missing). After excluding these participants, the final study sample comprised 611 of adolescents who reported their experiences for the last meeting they attended (age 11–16, *M*_*age*_ = 14.04, *SD* = 1.67, 47.1% female; 24.4% of all the sampled adolescents).

An external survey agency, STEM/MARK, handled sampling and data collection. Eligible participants were parents/caregivers who lived with an 11–16-year-old adolescent. Both the parent and the adolescent participated in the survey. The sample was recruited from a combined pool of three established online panels (i.e., ivyzkumy.cz, MNforce epanel, Kantar; together comprising approx. 165,000 panelists) and 980 newly recruited households. Quota sampling was used to ensure that the sample was proportionally representative of Czech households with children in terms of socioeconomic status (i.e., highest achieved education of the head of the household); region of residence based on the Nomenclature of Territorial Units for Statistics (NUTS3), which divides the country into 14 regions (see European Commission and Eurostat, 2020); and municipality size (five categories, 1 = less than 999 inhabitants – 5 = more than 100,000 inhabitants). Quotas were also used to achieve the equal representation of adolescents based on gender, age, and their combination.

The agency invited eligible panelists via e-mail. At the beginning of the survey, the parent provided demographic information. If they matched the set quota, they were asked to consent to their and the adolescent’s participation. The parent could look at the questions for the adolescents before providing their consent. Then they were instructed to call the adolescent and give them privacy. The adolescent’s questionnaire started with a brief introduction and asked for consent. After filling in their part, the adolescent returned the device to the parent to complete the parental survey. Parents could not access the adolescents’ replies, and vice versa. Each question included a response “Prefer not to answer”, which was treated as a missing response. As an incentive, each dyad received reward points redeemable for approx. €4 ≈ $4. The agency checked the data quality (i.e., questionnaire completion time, the consistency of the answers across the questionnaire, overall number of missing data). Only questionnaires that passed the quality checks were provided to the researchers. Researchers conducted additional data quality checks (e.g., checking response sets), and found no need to omit any questionnaires from the dataset. The survey was approved by the university’s ethical review board.

### Measures

All response scales included verbal anchors for each answer. For brevity, only the first and the last anchor are described here. The full wording of all of the items is available through Open Science Framework (OSF): https://osf.io/ub5sn/.

#### Participation in Face-to-Face Meetings

The participants were first given explanations for face-to-face meetings with people from the internet (i.e., *On the internet, people can have conversations with other people whom they do not know from real life - they have not met in person. These conversations can happen at various places (e.g., on social networks, in games, on dating sites, in internet discussions). We are not talking about “professional” communication (e.g., with e-shop, tutor, helpline). […] Some also meet people they know only from the internet face to face – in reality*.), and then asked how many such meetings they had participated in during their lifetime (*0* = *none – 4* = *four or more*) and how long ago had their last such meeting took place (*1* = *this year [in 2021] – 4* = *about three or more years ago [before 2019])*. From the 2,500 surveyed adolescents, 65.7% reported never having participated in a face-to-face meeting, 11.8% experienced one such meeting, 9.6% two, 3.8% three, 5.3% four or more, and 3.7% did not want to respond. Among those who experienced at least one meeting (*n* = 764), 21.6% experienced their last meeting in the year of data collection (2021), 39.7% in the preceding year, 18.7% about two years prior, 8.6% three or more years prior, and 11.4% did not want to respond. As explained in the Participants section above, the analysis included only the meetings that happened within the previous two years.

All of the following measures (except for the measure of social self-efficacy) asked about the adolescents’ most recent first-time meeting with someone they knew only from the internet.

#### Length of Prior Online Contact

Length of prior online contact was assessed by asking how long adolescents had been in touch with the person over the internet before they met face-to-face (*1* = *a couple of days or less – 5* = *for longer than six months*, *M* = 3.21, *SD* = 1.23, 2.6% missing).

#### Motives for Participation

Adolescents were asked why they wanted to meet someone from the internet using three yes/no items that represented three types of motives: romantic (*I wanted to go on a date, find myself a boyfriend/girlfriend*, 33.6% yes, 3.8% missing); friendly (*I wanted to have a chat with someone, get to know someone new*, 81.2% yes, 2.1% missing); and instrumental (*I wanted to get tutoring or exchange, sell, or buy something [e.g., collectibles, games, clothes]*, 44.5% yes, 1.6% missing). Since the adolescents could choose more than one motive, a variable with eight categories was constructed to correspond to all of the response combinations (i.e., 2.0% *romantic-only*, 33.4% *friendly-only*, 6.9% *instrumental-only*, 11.9% *friendly & romantic*, 16.2% *friendly & instrumental*, 1.8% *romantic & instrumental*, 17.0% *all three*, 5.7% *none*, 5.1% missing).

#### Pleasantness of the Meeting

One item was used to assess how pleasant the last meeting was for the adolescents (*And how was the meeting for you? 1* = *very unpleasant – 5* = *very pleasant*, *M* = 3.83, *SD* = 0.92, 0.8% missing).

#### Fear of Harm

One item measured how afraid the adolescents were of being harmed by the other person (*During the meeting, were you afraid that the person wanted to hurt you in any way? 1* = *definitely not – 4* = *definitely yes*), including a response that denoted uncertainty (*5* = *I am not sure*). Not enough adolescents chose the uncertainty response (*n* = 32, 5.2%) to include them as a specific group in the analysis. Thus, the response was treated as missing (*M* = 1.70, *SD* = 0.84, 5.9% missing).

#### Expectation Disconfirmation

To capture the disconfirmation of expectations with reality, adolescents were first asked about their expectations (*From what you knew about the person from the internet, who did you expect to meet face to face?*) about the person’s gender (*That they would be: 1* = *a girl/woman*, 49.8%*, 2* = *a boy/man*, 48.8%, 1.5% missing) and age (*That they would be: 1* = *a bit younger than me – 5* = *older* [than 30], *M* = 2.16, *SD* = 0.70, 0.7% missing). They were then asked who came to the meeting (*And who actually showed up to the meeting?*), using the same response options (gender: 48.9% female, 2.0% missing; age: *M* = 2.22, *SD* = 0.79, 0.8% missing). The responses were recoded into two variables that capture expectation disconfirmation of gender and age (*1* = *different, 2* = *as expected*, gender: 3.3% different, 2.9% missing; age: 15.9% different, 1.1% missing). Furthermore, two items measured the expectation disconfirmation of appearance and behavior of the person met on a five-point scale (*They looked / They behaved: 1* = *much worse than I had expected – 5* = *much better than I had expected)*. For easier interpretation, the items were recoded so that zero indicates that appearance/behavior of the person met matched the adolescent’s expectations (i.e., expectation confirmation), negative values indicate their appearance/behavior was worse than expected (i.e., negative disconfirmation), and positive values indicate their appearance/behavior was better than expected (i.e., positive disconfirmation) (appearance: *M* = 0.08, *SD* = 0.78, 1.3% missing; behavior: *M* = 0.27, *SD* = 0.85, 0.5% missing).

#### Continued Contact

Adolescents were asked whether they talked to the person or met them again after their first face-to-face meeting (*1* = *no, we met just once*, 22.9%*, 2* = *yes, we had met or talked, but we don’t anymore*, 31.3%*, 3* = *yes, we still meet or talk*, 44.4%, 1.5% missing). For easier interpretation, a dichotomized variable was used in the analysis (*1* = *no continued contact*, 22.9%, *2* = *some contact after meeting*, 75.6%).

#### Social Self-efficacy

Social self-efficacy was measured with four of the seven items that comprise the social self-efficacy subscale of the Self-efficacy Questionnaire for Children (Muris, 2001). The original items were generalized by replacing “classmates” with “peers” (e.g., *How easy or difficult has it been for you to: …express your opinions when other peers disagree with you?, …become friends with other people your age?*). The instructions were also shortened and the items and the response scale modified accordingly (*1* = *very difficult – 5* = *very easy*). The final score is an average across the items (*M* = 3.38, *SD* = 0.81, 0.3% missing). Confirmatory factor analysis (CFA) suggested that the scale was unidimensional (χ²(2) = 0.05, *p* = 0.973, CFI = 1.00, TLI = 1.01, RMSEA = 0.00 with 90% CI = [0.00, 0.00], SRMR = 0.00) and had good reliability (ω = 0.80).

### Analysis

The analysis was conducted with IBM SPSS v28.0.1.1 and with R v4.2.0, using the packages lavaan v0.6–12 for CFA and semTools v0.5–6 for reliability analysis.

First, face-to-face meetings were categorized based on the adolescents’ motivation to attend them, and the differences between these categories were examined. Meetings for which the adolescents reported that neither of the provided motives were true for them (*n* = 35) or did not respond to the questions (*n* = 31) were omitted. Differences in ordinal variables (i.e., adolescents’ age, length of prior online contact, expectations-reality congruence regarding the behavior and appearance of the person met, fear of harm), were assessed with Kruskal-Wallis tests and Dunn-Bonferroni post-hoc tests. Differences in categorical variables (i.e., adolescents’ gender, gender homophily, expectation disconfirmation regarding age, contact after meeting) were assessed with a chi-square test of independence with Bonferroni corrected z-tests. The expectation disconfirmation regarding gender was not examined because too few adolescents reported incongruence (*n* = 20 across all of the meetings) to warrant any substantiated conclusions.

Second, multinomial logistic regression was used to examine associations between adolescents’ motives for attending, social self-efficacy, and the expectation disconfirmation and pleasantness of the meeting, while controlling for adolescents’ age, gender, and the length of prior online contact. To achieve enough cases in each category of the outcome variable, the responses were collapsed into three categories: pleasant (i.e., responses *rather pleasant* and *very pleasant*; *n* = 387); neutral (i.e., response *neither pleasant nor unpleasant*; *n* = 117); and unpleasant (i.e., responses *rather unpleasant* and *very unpleasant*; *n* = 43). The same regression was run twice – first with *neutral* meetings as the reference category and then with *pleasant* meetings as the reference. This enabled a better examination of the differences between each pair of (unpleasant, neutral, pleasant) meetings.

The complete dataset and analysis scripts are available through OSF: https://osf.io/ub5sn/.

## Results

### Comparison of Face-to-Face Meetings Based on Motives

Among the seven categories of face-to-face meetings (based on adolescents’ motivation to attend them), the friendly-only motive were most frequent, followed by those with all three motives, friendly & instrumental motives, and friendly & romantic motives. By contrast, meetings attended with a romantic-only motive or romantic & instrumental motives were the least common. Table 1 shows the means and standard deviations of the ordinal variables, the proportions for the categorical variables for each category of the meetings, and the test results and effect sizes.

**Table 1:** Comparison of face-to-face meetings based on adolescents’ motives for participation

	Romantic (*n* = 12)	Friendly (*n* = 204)	Instrumental (*n* = 42)	Friendly & Romantic (*n* = 73)	Romantic & Instrumental (*n* = 11)	Friendly & Instrumental (*n* = 99)	All Three (*n* = 104)
**Ordinal Variables**							
Adolescent’s Age	*N* = 545, H(6) = 7.82, *p* = 0.252, ε² = 0.01						
*M* (*SD*), range 11 to 16	14.58 (1.08)	14.12 (1.66)	13.71 (1.69)	14.41 (1.42)	14.45 (1.97)	13.81 (1.83)	14.11 (1.68)
Length of Prior Online Contact	*N* = 533, H(6) = 19.08, *p* = 0.004, ε² = 0.04						
*M* (*SD*), range 1 to 5	3.08 (0.90) ^a, b^	3.44 (1.21) ^a^	2.77 (1.33) ^a, b^	3.14 (1.11) ^a, b^	2.91 (1.14) ^a, b^	3.22 (1.27) ^a, b^	2.98 (1.11) ^b^
Appearance Disconfirmation	*N* = 539, H(6) = 7.75, *p* = 0.257, ε² = 0.01						
*M* (*SD*), range −2 to 2	0.00 (0.95)	0.18 (0.66)	−0.12 (0.55)	0.10 (0.96)	0.00 (0.78)	0.02 (0.78)	0.06 (0.84)
Behavior Disconfirmation	*N* = 543, H(6) = 16.44, *p* = 0.012, ε² = 0.03						
*M* (*SD*), range −2 to 2	0.08 (1.08) ^a, b^	0.43 (0.81) ^a^	0.21 (0.61) ^a, b^	0.25 (0.93) ^a, b^	−0.45 (0.82) ^b^	0.27 (0.83) ^a, b^	0.17 (0.83) ^a, b^
Fear of Harm	*N* = 517, H(6) = 65.94, p = <0.001, ε² = 0.13						
*M* (*SD*), range 1 to 4	2.33 (1.15) ^b, c^	1.42 (0.63) ^a^	1.58 (0.76) ^a, b^	1.61 (0.82) ^a, b^	2.50 (0.53) ^c^	1.70 (0.85) ^a, b^	2.17 (0.96) ^c^
**Categorical Variables**							
Adolescent’s Gender	*N* = 545, χ²(6) = 22.99, *p* = 0.001, V = 0.21						
Female (%)	25.0 ^a, b^	59.3 ^b^	42.9 ^a, b^	49.3 ^a, b^	63.6 ^a, b^	39.4 ^a^	36.5 ^a^
Male (%)	75.0	40.7	57.1	50.7	36.4	60.6	63.5
Gender Homophily	*N* = 538, χ²(6) = 87.45, *p* < 0.001, V = 0.40						
Same-gender meeting (%)	33.3 ^a, b, c, d^	64.4 ^d^	90.0 ^e^	19.4 ^c^	18.2 ^a, b, c^	60.2 ^b, d^	35.0 ^a, c^
Cross-gender meeting (%)	66.7	35.6	10.0	80.6	81.8	39.8	65.0
Age Disconfirmation	*N* = 540, χ²(6) = 26.03, *p* < 0.001, V = 0.22						
Age as expected (%)	75.0 ^a, b^	91.6 ^b^	87.8 ^a, b^	84.9 ^a, b^	60.0 ^a^	84.8 ^a, b^	71.8 ^a^
Different than expected (%)	25.0	8.4	12.2	15.1	40.0	15.2	28.2
Contact After Meeting	*N* = 538, χ²(6) = 26.09, *p* < 0.001, V = 0.22						
No (%)	25.0 ^a, b, c^	17.0 ^c^	51.2 ^b^	23.3 ^a, b, c^	36.4 ^a, b, c^	17.3 ^a, c^	21.4 ^a, c^
Yes (%)	75.0	83.0	48.8	76.7	63.6	82.7	78.6

The table omits meetings where adolescents indicated no motive (*n* = 35) or did not answer (*n* = 31). Different letters in superscript indicate significant differences at α = 0.05. Differences were tested using Kruskal-Wallis tests with Dunn-Bonferroni post-hoc tests for ordinal variables and a chi-square test of independence with Bonferroni corrected z-tests for categorical variables

There were no significant differences among the categories of meetings in terms of the age of the adolescent and in the expectation disconfirmation regarding the appearance of the person met. In all of the other factors at hand, at least some categories of meetings differed from one another. For brevity, only the statistically significant differences are described further.

Meetings attended with friendly-only motives were significantly more often attended by female adolescents (59.3%) than meetings attended with friendly & instrumental or all three motives (39.4% and 36.5%, respectively). The reported online contact prior to the meeting was ongoing for significantly longer among friendly-only meetings than among meetings attended with all three motives.

The categories of meetings differed in the number that were with the person of the same or opposite gender (i.e., gender homophily). Almost all of the instrumental-only meetings were same-gender (90%), significantly more than any other category. Friendly-only meetings were also predominantly same-gender (64.4%), significantly more than meetings attended with romantic & instrumental, friendly & romantic, and all three motives. Similarly, meetings attended with friendly & instrumental motives were mostly same-gender (60.2%) and differed significantly from meetings attended with friendly & romantic or all three motives in this regard. All other categories of meetings (i.e., romantic-only, friendly & romantic, romantic & instrumental, and all three motives) were predominantly cross-gender.

The proportion of meetings where adolescents reported a mismatch between the expected and the real age of the person met was lowest among friendly-only meetings (8.4%). The category significantly differed from meetings attended with romantic & instrumental motives (where the proportion was highest – 4 out of 10) and with all three motives (second highest – 28.2%). Similarly, there was a significant difference in the behavior of the person met (in relation to the adolescent’s expectation) between friendly-only meetings and meetings attended with romantic & instrumental motives. Friendly-only meetings had the highest positive disconfirmation (i.e., the person behaved better than expected) of all of the categories, while romantic & instrumental motivated meetings had the highest negative disconfirmation (i.e., the person behaved worse than expected).

In friendly-only meetings, adolescents, on average, reported the lowest fear that the other person would harm them. They significantly differed from meetings attended with all three motives, romantic-only, or romantic & instrumental motives, where this fear was the highest. Instrumental-only, friendly & romantic, and friendly & instrumental meetings also had a significantly lower average fear of harm than meetings attended with all three motives or romantic & instrumental motives.

Lastly, the categories of meetings differed in how common it was for further contact to occur after the initial meeting. This was least common for instrumental-only motivated meetings (48.8%), which differed significantly from meetings attended with friendly-only, friendly & instrumental, or all three motives (83.0%, 82.7%, and 78.6%, respectively), where further contact was the most common.

### Factors Associated with Pleasantness of the Meeting

The results of multinomial logistic regression indicate which factors are associated with the likelihood that the meeting was rated as pleasant, neutral, or unpleasant by the adolescent (Table 2).

**Table 2:** Results of the multinomial logistic regression to predict the pleasantness of face-to-face meetings (*N* = 547)

	Pleasant (Reference Category: Neutral)				Unpleasant (Reference Category: Neutral)				Unpleasant (Reference Category: Pleasant)			
Predictors	*B* (*SE*)	*p*	*OR*	95% CI	*B* (*SE*)	*p*	*OR*	95% CI	*B* (*SE*)	*p*	*OR*	95% CI
Intercept	−1.95 (1.17)	0.095			−5.78 (2.04)	0.005			−3.83 (1.98)	0.053		
Gender (male)	0.14 (0.24)	0.554	1.15	[0.72, 1.83]	0.29 (0.40)	0.462	1.34	[0.61, 2.94]	0.15 (0.39)	0.691	1.17	[0.55, 2.50]
Age	−0.01 (0.07)	0.867	0.99	[0.86, 1.14]	0.21 (0.12)	0.092	1.23	[0.97, 1.57]	0.22 (0.12)	0.069	1.25	[0.98, 1.58]
Online Communication Length	**0.38 (0.10)**	**<0.001**	**1.47**	**[1.21, 1.78]**	−0.14 (0.18)	0.427	0.87	[0.62, 1.23]	−**0.52 (0.17)**	**0.002**	**0.59**	**[0.43, 0.83]**
Social Self-efficacy	**0.38 (0.15)**	**0.011**	**1.46**	**[1.09, 1.95]**	0.30 (0.26)	0.232	1.36	[0.82, 2.23]	−0.07 (0.25)	0.773	0.93	[0.57, 1.51]
Motives^a^												
Romantic	0.14 (0.26)	0.599	1.15	[0.69, 1.90]	0.71 (0.41)	0.084	2.03	[0.91, 4.54]	0.57 (0.40)	0.148	1.77	[0.82, 3.85]
Friendly	**1.10 (0.28)**	**<0.001**	**3.00**	**[1.72, 5.23]**	−0.40 (0.44)	0.364	0.67	[0.29, 1.58]	−**1.49 (0.44)**	**0.001**	**0.23**	**[0.10, 0.53]**
Instrumental	−0.38 (0.24)	0.119	0.69	[0.43, 1.10]	−0.16 (0.41)	0.703	0.86	[0.38, 1.92]	0.22 (0.40)	0.580	1.25	[0.57, 2.72]
Expectation Disconfirmation^b^												
Gender	−0.65 (0.69)	0.353	0.53	[0.14, 2.05]	1.16 (0.71)	0.101	3.18	[0.80, 12.66]	**1.80 (0.76)**	**0.017**	**6.06**	**[1.38, 26.69]**
Age	0.06 (0.33)	0.869	1.06	[0.55, 2.03]	0.86 (0.44)	0.052	2.35	[0.99, 5.58]	0.80 (0.43)	0.063	2.23	[0.96, 5.17]
Appearance (positive)	0.21 (0.37)	0.565	1.24	[0.60, 2.54]	0.38 (0.65)	0.564	1.46	[0.41, 5.26]	0.17 (0.59)	0.779	1.18	[0.37, 3.78]
Appearance (negative)	−**0.85 (0.36)**	**0.017**	**0.43**	**[0.21, 0.86]**	0.69 (0.48)	0.149	1.99	[0.78, 5.08]	**1.54 (0.48)**	**0.001**	**4.67**	**[1.83, 11.92]**
Behavior (positive)	**0.88 (0.31)**	**0.005**	**2.42**	**[1.31, 4.45]**	1.02 (0.56)	0.067	2.76	[0.93, 8.19]	0.13 (0.51)	0.794	1.14	[0.42, 3.07]
Behavior (negative)	−**0.82 (0.40)**	**0.041**	**0.44**	**[0.20, 0.97]**	0.97 (0.51)	0.057	2.63	[0.97, 7.11]	**1.79 (0.52)**	**0.001**	**5.99**	**[2.17, 16.56]**

Likelihood ratio test: χ² (26) = 166.39, *p* < 0.001; Pseudo R^2^: Cox & Snell = 0.26, Nagelkerke = 0.33. Results significant at α = 0.05 are in bold

^a^Reference category: *No* (i.e., motive absent)

^b^Reference category: *As expected* (i.e., expectation confirmation)

First, the likelihood that the meeting was pleasant, rather than neutral, was examined. Adolescents with higher social self-efficacy and those who communicated online for longer were more likely to report a pleasant meeting than a neutral one. Attending with a friendly motive increased the likelihood of a pleasant meeting, while other motives had no effect. Regarding expectation disconfirmation, when the person met behaved better than expected (i.e., positive disconfirmation), the meeting was more likely to be pleasant. When their behavior or appearance was worse than expected (i.e., negative disconfirmation), the meeting was less likely to be reported as pleasant. There was no effect for better-than-expected appearance or a mismatch in age or gender.

Second, the likelihood of the meeting being unpleasant, rather than neutral, was examined. None of the predictors significantly related to this likelihood. This may have been because of the low statistical power caused by the lower number of unpleasant and neutral meetings. Despite not being significant, some results indicate medium-sized effects (*OR* > 2, *OR* < 0.5) with relatively low p-values (*p* < 0.101). These suggest that the dating motive, mismatch in age or gender, worse-than-expected appearance or behavior, and better-than-expected behavior all related to a higher likelihood for the rating of the meeting to be unpleasant.

Lastly, to overcome issues with low power, the likelihood that the meeting was unpleasant, rather than pleasant, was examined. Longer online communication and the presence of social motive decreased the likelihood of a negative meeting. When the person behaved or looked worse or was of a different gender than expected, the meeting was more likely to be negative. Different-than-expected age and better-than-expected appearance or behavior did not relate to the likelihood of adolescents rating the meeting as either pleasant or unpleasant. Adolescents’ age, gender, social self-efficacy, and other motives also had no effect.

### Alternative Models

Initially, relationships between predictors and the pleasantness of the meeting were tested using an ordinal regression. However, the test of parallel lines suggested that the assumption of proportional odds was violated (χ² (42) = 73.12, *p* = 0.002). A sequence of binomial regressions at each cumulative threshold showed large differences in the odds ratios. Thus, ordinal regression was not suitable and multinomial logistic regression was used instead. Three versions of the multinomial regression were tested. In Model 1, social self-efficacy was not included and the variable to capture expectation disconfirmation in age had three categories (i.e., *age as expected, person younger than expected, person older than expected*). In Model 2, the age-disconfirmation variable was collapsed into two categories. In Model 3 (reported above), social self-efficacy was included. Apart from a negligible variation in the size of the odds ratios, the overall pattern of results remained equivalent across the three models. The only differences were in the effects of the age disconfirmation, which were not significant in Model 3 (reported in Table 2). In Model 1, the person being younger than expected increased the likelihood of an unpleasant (rather than a pleasant) meeting (*OR* = 4.03, *p* = 0.030). In Model 2, the person being of a different age than expected increased the likelihood of an unpleasant (rather than a neutral) meeting (*OR* = 2.43, *p* = 0.042).

## Discussion

The existing body of research about in-person, face-to-face meetings between adolescents and people they met on the internet is founded on the assumption that these meetings are motivated by the desire to form new social relationships. This study aimed to test this assumption by categorizing the meetings based on the adolescents’ motives. There were significant differences in most of the considered factors (which are detailed in the first part of the Discussion), which support the idea that it is necessary to differentiate between the types of face-to-face meetings in both research and public discourse. Moreover, while previous studies examined how often these meetings are pleasant or unpleasant for adolescents, the factors and mechanisms that may explain adolescents’ experiences were largely left untested. The present findings suggest that the pleasantness of the meeting is closely related to (dis)confirmation of the adolescents’ expectations about the person they met. Furthermore, meetings attended with friendly motives and those preceded by a longer period of online contact were more likely to be pleasant and less likely to be unpleasant.

### Different Motives, Different Meetings

The results of this study indicate that four out of five adolescents participated in a face-to-face meeting with someone from the internet at least partly with a friendly motive (i.e., to meet someone new, make a new friend). The frequent presence of a friendly motive supports the assumption that adolescents use online communication primarily to expand their social circle, which underlies both the social compensation and the rich-get-richer hypotheses (Mesch, 2019), and the studies that rely on those hypotheses (e.g., Bayraktar et al., 2016). However, every fifth adolescent goes to the meeting with other motives (i.e., specifically to find a girlfriend/boyfriend, for instrumental purposes like to buy or sell something, get tutoring) or for some other reason not captured in this study. For these types of meetings, the aforementioned theories might need to either be adapted or replaced by more fitting ones. Thus, future research, especially when driven by hypotheses that presume socially motivated interactions, should carefully differentiate between the motives for such interactions.

The need to differentiate between meetings attended with varying motives is emphasized by the differences within these types of meetings. Based on the results, the seven examined categories of meetings can be sorted into three types that seems to be structurally more similar – friendly-only meetings; meetings with instrumental component (i.e., instrumental-only, instrumental & friendly); and meetings with romantic component (i.e., romantic-only, friendly & romantic, romantic & instrumental). Friendly-only meetings were by far the most common in this sample. In comparison to the other categories, online contact prior to these meetings was the longest and, most adolescents stayed in touch with the person after friendly-only meetings. Meeting someone who is unexpectedly older or younger was the rarest occurrence for these meetings and, on average, the person met behaved better than expected and the adolescents had the lowest level of fear.

Compared to friendly-only meetings, meetings with instrumental component are more often attended by male adolescents. At these meetings, adolescents also mostly meet with someone of the same gender. However, instrumental meetings are not completely uniform. The proportion of same-gender meetings is much higher for the instrumental-only motive (significantly higher than in any other category). Most adolescents stay in touch with the other person after a friendly & instrumental meeting, but less than half stay in touch after an instrumental-only meeting. Thus, instrumental-only meetings seem to be mostly one-off encounters that are focused on the instrumental goal (e.g., buying/selling something), while friendly & instrumental meetings represent encounters where adolescents bond over shared interest (e.g., exchanging collectibles). Considering the higher participation of male adolescents, the present findings fit the research that shows that female adolescents spend more time using social media and texting and male adolescents spend more time gaming (Twenge & Martin, 2020). This suggests that, when female adolescents form new relationships over the internet, they tend to rely on interpersonal communication, while male adolescents rely on shared activities and hobbies.

Meetings that had some romantic component are more often cross-gender than other meetings. Assuming that most adolescents are heterosexual, this result is not novel, but it supports the plausibility for the presented categorization of face-to-face meetings. Friendly & romantic meetings, which are the most common type of romantic meetings, do not significantly differ from other types of meetings in any other aspect. However, adolescents who attended romantic-only meetings felt more afraid that the person they were meeting would harm them (compared to friendly-only meetings). This is even more pronounced for romantic & instrumental meetings, where adolescents’ fear of harm is higher than in most other meetings (including friendly & romantic ones). Moreover, encountering someone who was of a different age or who behaved worse than expected was more common at romantic & instrumental meetings, than at friendly-only ones. These results are in line with a qualitative study that showed that upsetting meetings were mostly romantically-motivated and the adolescents who attended them reported that the other person made unwelcome attempts at physical contact or behaved in an aggressive or overconfident manner (Dedkova et al., 2014) (i.e., behaved worse than expected). The higher rate of age disconfirmation supports the notion that misrepresenting one’s age may be more common in dating as part of strategic misrepresentation (Huang & Yang, 2013). Overall, the comparatively high levels of the fear of harm and expectation disconfirmation indicate that the romantic & instrumental meetings (and, to a lesser degree, the romantic-only ones) have a higher risk potential. It is necessary to emphasize that only a few adolescents in the currently analyzed sample reported these types of meetings, which complicates any generalizations. It is also hard to interpret the specific situations that are represented with the combination of romantic and instrumental motives. Future research should focus on a deeper understanding of romantically motivated face-to-face meetings, and testing whether they are indeed more risky than other meetings.

Meetings attended for all three examined motives stand apart from the above-mentioned three main groups of meetings as it is less clear what their dominant “driving force” is. In several aspects, these meetings resemble those with romantic component (they are mostly gross-gender, age disconfirmation is more common, fear of harm is higher). However, they also differ from other meetings in ways that meetings with romantic component do not – they are more frequently attended by male adolescents and prior online contact is shorter compared to friendly-only; further contact after the meeting is more common than for instrumental-only meetings. Meetings attended for all three represent substantially large category, which warrants more detailed examination in future studies.

The aforementioned results demonstrate that going to a face-to-face meeting with someone from the internet is far from a uniform experience. Nevertheless, the results support some more general conclusions. Despite the common fear of deceptive online predators (boyd et al., 2009), the current results show that adolescents usually met whom they expected. This confirms and expands upon the results from the Netherlands, where the mismatch between expectations and reality was also rare (Van den Heuvel et al., 2012). Only a few studies focused on this topic, and they are quite outdated (a decade old). The current study provides evidence that the changes in ICT landscape (e.g., more prevalent usage of smartphones generally as well as location-based dating apps specifically) did not substantially shift the occurrences of misrepresentation of one’s identity.

Except for romantic & instrumental meetings, the appearance and behavior of the person mostly conforms to the adolescents’ expectations. Any disconfirmations were more likely to be positive (i.e., the person behaving and looking better) than negative. Consistent with previous studies (e.g., Smahel et al., 2020), meeting someone from the internet is usually a pleasant experience and unpleasant meetings are relatively rare. Similarly, in most types of meetings, adolescents seldom feel afraid that the person would harm them. Together with a previous study that showed that sexual abuse by people met online is less prevalent than by people met elsewhere (Priebe & Svedin, 2012), the current results challenge the widespread notion that meeting people from the internet increases the likelihood of sexual or other abuse (see Holmes, 2009; Mascheroni et al., 2014). Instead, this study suggests that, rather than a threat, these meetings are an opportunity to make new social connections. Not only are the meetings predominantly pleasant, but, for most meetings (especially friendly motivated ones), the relationships continue after the meeting. Cernikova et al. (2018) showed that, on the internet, children and adolescents evaluate new people (e.g., based on SNS profile), select those with whom they want to communicate, and continue their evaluation during online communication. When the interaction is negative, they discontinue it. Thus, people considered for an in-person meeting, had already “passed” several evaluation thresholds. This contributes to the overall high proportion of pleasant experiences.

### Meeting Pleasantness and Adolescents’ Expectations

Consistent with the expectation-disconfirmation theory (Oliver, 1980), an incongruence between adolescents’ expectations and the real characteristics of the people met closely relates to how pleasant the meeting was for adolescents. Disconfirmed expectations about gender or age increase the likelihood of an unpleasant meeting (rather than a pleasant or neutral one) but it does not relate to the likelihood of a pleasant meeting (rather than a neutral one). In other words, disconfirmation of expectations related to demographics can make a meeting less pleasant, but their confirmation alone does not make the meeting more pleasant. Negative disconfirmation of adolescents’ expectations about the behavior or appearance of the other person works similarly: unmet expectations are associated with unpleasant meetings. This is in line with a qualitative study that identified deviations from expectations as the primary reason that adolescents did not enjoy face-to-face meetings (Dedkova et al., 2014). As expected, behavior-related positive disconfirmation (i.e., situations where the demeanor of the person met exceeded expectations) was associated with pleasant meetings (better-than-expected appearance had no effect). This validates the usage of the expectation-disconfirmation theory outside of its original context (i.e., consumer satisfaction). The present findings demonstrate that the pleasantness of a face-to-face meeting largely depends on adolescents’ expectations. Thus, when adolescents rate a meeting as unpleasant, it does not necessarily imply harm or abuse. It might be because the person did not meet the adolescents’ expectations. In the cases of age and gender, expectation disconfirmation is likely caused by deception. However, as proposed by the *hyper-personal model*, computer-mediated communication can lead to exaggerated perceptions and unrealistic expectations about the other person (Walther, 1996; Walther & Whitty, 2021). Therefore, unmet expectations about behavior (and possibly appearance) may be caused by the specific features of online communication. Future research should investigate adolescents’ expectations in more detail to see whether they tend to be realistic.

According to the analysis, adolescents with higher social self-efficacy are more likely to rate meetings as pleasant rather than neutral, but social self-efficacy does not relate to the likelihood of the meeting being unpleasant. Thus, adolescents can use their social skills to make the meeting more pleasant, but these skills do not protect them from negative experiences. This is not to say that social self-efficacy is not important. In this study, unpleasant meetings likely included situations where no severe harm was done. In situations where harm occurred, social skills could play a protective role or they could be important in coping with the aftermath (Bijstra & Jackson, 1998).

Presented analysis suggests that when online contact is longer, adolescents are more likely to rate the meeting as pleasant. Perhaps, longer online contact allows adolescents to better assess their online acquaintances and then meet only those whom they like. Adolescents may also develop a stronger bond through longer online contact. Since they already know the person, the resulting meeting may be less awkward and more enjoyable. Though more research about the beneficial role of longer online contact is necessary, getting to know the person better through online conversations that span a longer time-period before meeting them in person can be recommended.

### Limitations and Future Directions

This study used data from a general sample of Czech adolescents. Consequently, there were relatively few cases of the rarer types of meetings (e.g., unpleasant ones, meetings with a romantic-only motive), despite the large initial sample size. While this approach allowed us to assess the prevalence of various types of meetings, the results pertaining to the rarer meetings should be generalized with caution. Future research should specifically focus on adolescents who experienced face-to-face meetings or oversample the uncommon experiences.

While this study was the first to directly explore the motives for participation, it relied on a broad categorization. A qualitative approach could identify more nuanced motives and illuminate the dynamics behind some of the less common combinations of motives found in the study. In quantitative research, it might be fruitful to ask about the adolescents’ primary motives. Future research could also consider the specific online environments (e.g., social networking sites, dating applications) where adolescents meet their partners or investigate face-to-face meetings with multiple people.

Lastly, the study focused on meetings that happened between 2019 and 2021. This included a period of time during the COVID-19 pandemic. Contact restrictions may have precluded adolescents from meeting online acquaintances face-to-face. By contrast, limited contact with schoolmates caused by periods of online education may have motivated adolescents to compensate by meeting people from the internet. Such dynamics must be considered because they may have influenced the characteristics of the examined meetings.

## Conclusion

It is not unusual for adolescents to meet in person with someone they know only from the internet. While such face-to-face meetings encompass a variety of distinct situations, they are usually studied as a uniform phenomenon. The current study demonstrates that face-to-face meetings meaningfully differ from each other based on the adolescents’ motives for attending them. On one hand, current findings lend credibility to previous studies that built on the assumption that adolescents attend face-to-face meetings primarily to make new social connections. On the other hand, in one fifth of the face-to-face meetings, adolescents’ participation is driven by different motives (e.g., romantic, instrumental). Given the differences between meetings attended for different motives, it is important to separate different types of face-to-face meetings in future research and public discourse. This study also shows that the pleasantness of a face-to-face meeting is closely related to adolescents’ expectations about the person they are meeting. This demonstrates the utility of the expectation-disconfirmation theory for the investigations of interpersonal interactions and suggests that, when a face-to-face meeting is labeled as unpleasant, it does not have to mean that it was harmful for the adolescent.
