# Correction: Czech Adolescents’ Face-to-Face Meetings With People from the Internet: The Role of Adolescents’ Motives and Expectations

**DOI:** 10.1007/s10964-022-01730-1

**Published:** 2022-12-30

**Authors:** Vojtěch Mýlek, Lenka Dedkova, Gustavo S. Mesch

**Affiliations:** 1 Interdisciplinary Research Team on Internet and Society, Faculty of Social Studies, Masaryk University, Joštova 10, Brno, 60200 Czech Republic; 2 Department of Sociology, Faculty of Social Sciences, University of Haifa, 199 Aba Khoushy Ave. Mount Carmel, Haifa, 3498838 Israel

Correction: Journal of Youth and Adolescence (2022)

10.1007/s10964-022-01697-z

The article “Czech Adolescents’ Face-to-Face Meetings With People from the Internet: The Role of Adolescents’ Motives and Expectations”, written by Vojtěch Mýlek, Lenka Dedkova and Gustavo S. Mesch, was originally published electronically on the publisher’s internet portal on 8 November 2022 without open access. With the author(s)’ decision to opt for Open Choice the copyright of the article changed on 19 December 2022 to © The Author(s) 2022 and the article is forthwith distributed under a Creative Commons Attribution 4.0 International License, which permits use, sharing, adaptation, distribution and reproduction in any medium or format, as long as you give appropriate credit to the original author(s) and the source, provide a link to the Creative Commons licence, and indicate if changes were made. The images or other third party material in this article are included in the article’s Creative Commons licence, unless indicated otherwise in a credit line to the material. If material is not included in the article’s Creative Commons licence and your intended use is not permitted by statutory regulation or exceeds the permitted use, you will need to obtain permission directly from the copyright holder. To view a copy of this licence, visit http://creativecommons.org/licenses/by/4.0.

The original article has been corrected.

